# Supplementary material for: Mesenchymal Stem Cell Therapy in Alopecia Areata: Visual and Molecular Evidence from a Mouse Model
Source: Int J Mol Sci. 2024 Aug 26;25(17):9236. doi: 10.3390/ijms25179236 (PMC11394813; doi:10.3390/ijms25179236)
Supplement: Supplementary file 1 [file ijms-25-09236-s001.zip › ijms-3062755-supplementary.pdf]

**Supplementary Figure S1: Gross images of the dorsal skin of all 35 AA-induced mice from week 0 to week 10**

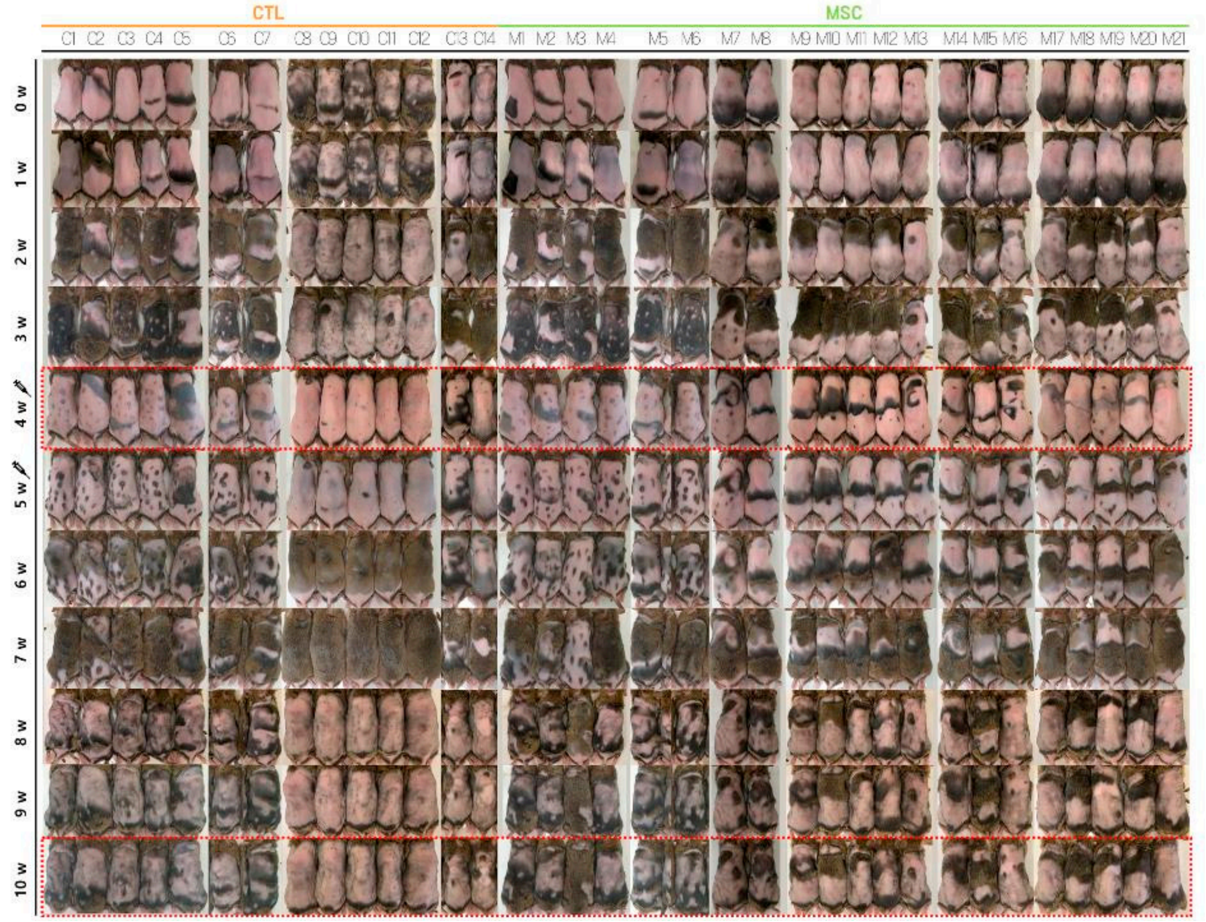


Mice C1–C14 belong to the CTL group, and mice M1–M21 belong to the MSC group. At week 4, extensive AA patches were observed in both groups. In the MSC group, diluted cultured hMSCs were intradermally injected into eight dorsal sites at week 4 and 5, while saline was injected in the CTL group during the same period. By week 7, hair regrowth was evident in most mice from both groups. However, hair loss recurred during the subsequent hair cycle, with prominent hair loss observed in most mice from both groups at week 8, and continuing through week 10.
